# A Case of Rabies in a Boy Nine Years Old

**Published:** 1917-02

**Authors:** Francis E. Fronczak

**Affiliations:** Health Commissioner, Buffalo, N. Y.


### A Case of Rabies in a Boy Nine Years Old.

By DR. FRANCIS E. FRONCZAK,

Health Commissioner, Buffalo, N. Y.

The following is the history of a case of rabies in a human being as obtained from the family.

T.......... C.........., age 9, residence Buffalo.

Bitten by a small black and white dog on Thursday, 6 p. m., six weeks ago.

Was walking on the street when the dog ran up to him, snuffed his feet and bit him on the hand.

The boy's father took him to a physician's office, the physician cauterized the wound and informed the father it would be all right. No other medical care given.

The boy remained well until Tuesday, January 9th, when he presented the following symptoms:

On arising in the morning, he looked extremely pale, had a peculiar staring expression and complained of not feeling well. Only drank coffee for his breakfast. On returning from school at noon, laid down most of the time. On returning from school in the afternoon, again laid down, saying he did not feel well. During the evening, he was unusually quiet, indisposed to activity and depressed, and slept poorly during the night.

Wednesday—on arising, looked very badly with staring eyes and startling look, complained of feeling badly and of pain in his hand where he was bitten. Ate little or nothing and remained home from school.

P. M.—General appearance the same, was given a drink of water which, however, he could not swallow on account of intense spasms of the throat, when the physician was sent for. On this first call, the doctor did not say definitely what the trouble was, and he was not informed about the inability to swallow. During the rest of the day and night, the symptoms increased with complete inability to swallow and extreme fright at the sight of water; boy extremely restless and passed a sleepless night.

Thursday—Physician called in the middle of the day and said the boy was nervous and possibly his condition was due to the dog bite, and changed the medicine. During the afternoon all symptoms became intensified, and during the night he suffered a great deal with spasms and difficulty on attempting to swallow, had fever and heavy heart action. Some delirium was present, the boy fancying he saw peculiar things—no sleep.

Friday—All symptoms aggravated, and, in addition, could not walk (paralysis). Physician called and recommended his removal to the hospital and repeated, the sickness was in consequence of the dog biting. A second physician was sent for, who at once inquired if the child had been bitten by a dog, recognized the condition and informed the family it was too late. Advised removal to the hospital, and that he would send for special treatment as a last resort (Pasteur). During the night the child's condition was serious, all symptoms being intensified.

Saturday—Physician called and informed the family that nothing could be done and the boy would soon die. The boy was removed, however, to the hospital at one o'clock and died that night.

—

The rule of the Department of Health is to secure the administration of the Pasteur treatment in all cases of biting by unknown dogs, and under all instances where it is not definitely ascertained that the dog is not rabid. In the case in question, through inadvertance or otherwise, no report of the accident was made either to the Police or Health Department, in accordance with the well known regulations in regards to same, the result was the development of the malady after the usual period of incubation.

The unfortunate case is of extreme interest in that it is the first one to occur in a human being in the city in very many years, and that the symptoms were characteristic (pain in the wound, anxious expression, restlessness, laryngeal spasm and dread of water, delirium and later paralysis—a picture which, together with the history, eliminates any other malady).

The incident also serves to illustrate the importance of reporting all cases of dog bites to the Police or Department of Health promptly in order that they may be investigated and the possible development of rabies averted. The department's procedure under these circumstances consists in securing and confining all offending animals at the Pound until the existence of rabies can be definitely eliminated or when, on the contrary the animal has or develops the malady, provision can be made for the Pasteur treatment.

---

**Wassermann Reaction on Tuberculosis.** Captains C. A. Shaw and A. T. Cooper, Am. Jour. Med. Sci., Vol. 152, No. 2, studying 290 cases of tuberculosis, conclude that if non-cholesterinized antigens are used, a double plus reaction is as significant of syphilis in a tubercular individual as in any one else but that if cholesterinized antigens are used, tuberculous persons free from syphilis, give a reaction varying from doubtful to double plus in 31%.

